# Recalibrating the ‘world map’ of palliative care development

**DOI:** 10.12688/wellcomeopenres.15109.2

**Published:** 2019-08-16

**Authors:** Nicole Baur, Carlos Centeno, Eduardo Garralda, Stephen Connor, David Clark

**Affiliations:** 1 School of Interdisciplinary Studies, University of Glasgow, Dumfries Campus, Dumfries, DG1 4ZL, UK; 2 Institute for Culture and Society, Health Research Institute of Navarra (IDISNA), University of Navarra, Pamplona, 31009, Spain; 3 Worldwide Hospice and Palliative Care Alliance, Fairfax, Virginia, 22039, USA

**Keywords:** Palliative care, global development, hospice, mapping

## Abstract

**Background**: Despite growing interest from policy makers, researchers and activists, there is still little science to underpin the global development of palliative care. This study presents the methods deployed in the creation of a ‘world map’ of palliative care development. Building on two previous iterations, with improved rigour and taking into account reviewers’ feedback, the aim of the study is to determine the level of palliative care development in 198 countries in 2017, whilst ensuring comparability with previous versions. We present methods of data collection and analysis.

**Methods and analysis:** Primary data on the level of palliative care development in 2017 was collected from in-country experts through an online questionnaire and, where required, supplemented by published documentary sources and grey literature. Population and per capita opioid consumption data were derived from independent sources. Data analysis was conducted according to a new scoring system and algorithm developed by the research team.

**Ethics and dissemination: **The study was approved by the University of Glasgow College of Social Sciences Research Ethics Committee. Findings of the study will be disseminated in peer-reviewed journals, as a contribution to the second edition of the Global Atlas of Palliative Care at the End-of-Life, and via social media, including the Glasgow End of Life Studies Group blog and the project website.

**Limitations of the study: **There are potential biases associated with self-reporting by key in-country experts. In some countries, the identified key expert failed to complete the questionnaire in whole or part and data limitations were potentially compounded by language restrictions, as questionnaires were available only in three European languages. The study relied in part on data from independent sources, the accuracy of which could not be verified.

## Introduction

The need for palliative care is increasing around the world and has recently been estimated at over 61 million patients per year ^1^. The World Health Assembly has called on all its member states to develop strategies to integrate palliative care into policies for health and social care across the lifespan ^2^. Whilst interest in the global development of palliative care is growing, there remains little science to underpin it. 

**Figure 1.**
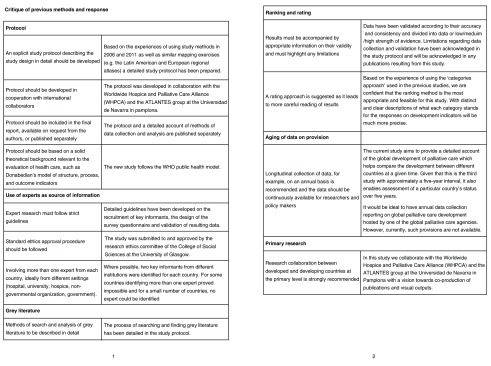
Critique of previous methods.

In an effort to advance methodological understanding of the challenges in conducting research on global palliative care development and how they may be overcome, we present here the methods deployed in producing a third ‘world map’ of palliative care development, allocating 198 countries to one of six categories, based on 10 key indicators. The chosen method for the study builds on previous iterations of the world map but is significantly improved in rigour and replicability. It also takes into account feedback and commentary received on previous versions from within the global palliative care community and from a small number of published works ^3, 4^ ( Figure 1). The improvements made to the methodology lead us to a recalibration of the world map. Comparability with the results of the earlier studies has been an enduring consideration, although not at the expense of improving the underlying method.

There is growing engagement from policy makers, researchers and activists in the development of palliative and end-of-life care in a global context ^5– 8^. This builds on discussions about how palliative care began as a social and medical movement in the West and over time expanded its scope and influence ^9^. Palliative care has been recognized as a public health issue ^10^. Closely linked to this, palliative care availability and access have also been acknowledged as a matter of ‘human rights’ in recent years ^11^, in a context where 80% of the need for palliative care is in low and middle-income countries. At the same time, comparative research on palliative care between countries remains challenging due to enormous inequity in the provision of services ^12^ as well as varying policy contexts and implementation practices ^13^. Comparative data about the distribution of services remain difficult to obtain, yet these are vital in informing global palliative care policy and in monitoring policy impacts.

Since 2006, the last author (DC) has been engaged in efforts to categorize palliative care development country by country throughout the world and to depict this development in a series of world and regional maps. 

For the first ‘world map’ ^14^, data about palliative care development from 2006 were collected from published articles in peer-reviewed and professional journals, books and monographs, palliative care directories and related websites, relevant reviews and databases, grey literature and conference presentations. These were then synthesised in order to allocate each country to one of the categories of development. Expert opinion was also used as a substitute for documentary sources where these were not available to inform the classification. The methods were weak, but nevertheless allowed a first foray into research of this scope and scale.

The four-part typology categorisation for the first world map was initially constructed for a review of palliative care in Africa ^15^. The four categories were:

(1) No identified hospice-palliative care activity
(2) Capacity building activity but no service
(3) Localized palliative care provision
(4) Palliative care activities approach integration with mainstream service providers

The second world map ^16^ involved a more systematic approach to the identification of in-country experts or ‘champions’ who were asked to give an opinion on the level of development of palliative care in their country in 2011, based on a refined six category classification:

(1) No known hospice-palliative care activity
(2) Capacity-building activity
(3a) Isolated palliative care provision
(3b) Generalized palliative care provision
(4a) Countries where hospice-palliative care services are at a stage of preliminary integration into mainstream service provision
(4b) Countries where hospice-palliative care services are at a stage of advanced integration into mainstream service provision.

The palliative care ‘champions’, with their extensive knowledge of both national and international development, were identified from published and grey documentary sources of information provided by 66 national palliative care associations and international palliative care agencies (International Association of Hospice and Palliative Care, Help the Hospices, Worldwide Hospice Palliative Care Alliance). Where no palliative care ‘champion’ could be identified for a country, regional palliative care associations (Asia Pacific Hospice Palliative Care Network, African Palliative Care Association, European Association for Palliative Care, Latin American Palliative Care Association) acted as ‘proxies’ for named countries within their jurisdiction. Where this was not possible, published and grey literature, and data contained in regional palliative care ‘atlases’ from Europe and Latin America which had appeared since the publication of the first world map, also provided material on which the researchers made a classification. Data from the second world map were in turn used to support the production of the first global atlas of palliative care, published jointly by the Worldwide Hospice Palliative Care Alliance and the World Health Organization ^17^.

The protocol and methods reported here for the third iteration of the world map of palliative care development build on the earlier work but go significantly beyond it. In a major departure from the earlier studies, the third world map is based primarily on an online survey of in-country experts, in which the questionnaire they were asked to complete was constructed to measure levels of development against 10 indicators drawn from the emerging literature. Where these data could not be obtained or were incomplete, they were enhanced by systematic searches of the published and grey literature for the countries in question. In addition, data on levels of opioid consumption, a well-established indicator of the level of palliative care development and delivery, and population were taken for all countries from reliable global sources. Additionally, the included jurisdictions were restricted to those 193 member and 2 observer states of the United Nations (UN), along with Kosovo, Somaliland, and Taiwan, China. This therefore excluded other associated territories, some of which had been included in previous iterations.

## Protocol

The present study, to produce a third iteration of the world map of palliative care development, forms part of a Wellcome Trust Investigator Award. It has benefitted from greater funding support than previous iterations and from fuller collaboration between project partners.

The objectives of the study are:

1) To establish the level of palliative care development in UN countries in 2017.
2) To implement methodological improvements, based on recent progress in the field and commentaries relating to the previous ‘world map’ studies.
3) To monitor development in global palliative care since 2006 and 2011.

### Recruitment and sampling of participants

For the third world map, we sought to recruit at least two palliative care expert respondents from each country (n=198). For this we created a database of key informants in collaboration with our project partners Worldwide Hospice Palliative Care Alliance (WHPCA) and ATLANTES research group (Universidad de Navarra). This enabled us to make use of published named contacts in countries where regional mapping of palliative care had been undertaken, i.e. Africa, Europe, Latin America and the Eastern Mediterranean region, as well as to capitalise on the partners’ wider networks. We also worked with the regional palliative care associations African Palliative Care Association (APCA), Latin American Association of Palliative Care (ALCP), Asia Pacific Hospice Palliative Care Network (APHN) and European Association for Palliative Care (EAPC), as well as the International Association for Hospice and Palliative Care (IAHPC), to identify key experts in areas where no atlas or mapping had taken place. Further contact details were obtained through authorship of peer-reviewed articles as well as posts and recruitment calls on social media. Names, workplaces and contact details of experts were collated in an electronic database and organised by World Health Organization (WHO) region and country.

Based on this database, sampling then took place according to the following algorithm, which listed preferred categories of informant in declining order of preference:

I) Representatives of the national in-country hospice-palliative care association or nearest professional association (e.g. society for palliative medicine or hospice forum). The person should have an established administrative and/or leadership role in the organisation, making them a reliable source of information. They will also play an important part in identifying further key informants within the country.
II) Academic experts with known interests and research experience in hospice-palliative care development in-country and/or beyond, as evidenced by peer-reviewed publications. The person should have an established academic role in hospice-palliative care research or education, making them a reliable source of information.
III) Policy specialists (in or outside government) with experience of and/or responsibility for hospice-palliative care delivery in-country. The person should have an established policy role relating to hospice-palliative care, making them a reliable source of information.
IV) Palliative care representatives, academics or policy specialists from outside the country but with direct knowledge of its hospice-palliative care provision, making them a reliable source of information.

560 contacts were identified across 179 countries. Their distribution can be seen in Table 1. Despite best efforts, we were unable to identify contacts for the remaining 19 countries.

**Table 1.** Numbers of contacts per country.

No of contacts per country	No of countries
Countries with 1 contact	33
Countries with 2 contacts	48 ^i^
Countries with 3 contacts	37
Countries with 4 contacts	26
Countries with 5 contacts	16
Countries with 6 contacts	4
Countries with 7 contacts	8
Countries with 8 contacts	2
Countries with 9 contacts	2
Countries with ≥ 10 contacts	3

^i^Figure excludes Timor L’este where originally two contacts were identified, but both did not feel that they were the right people to complete the questionnaire, i.e. we had no contact in Timor L’este

### Data collection

Data collection focussed primarily on the questionnaire which in-country contacts were asked to complete. This was supplemented where required, by published documentary sources and grey literature. Opioid consumption and population data came from independent sources, as explained below.

***Online questionnaire***. Between September 2017 and January 2018, a questionnaire was developed collaboratively by the project team (For a copy of the questionnaire see Extended data) ^18^. In January and February 2018, the questionnaire was piloted for content by 8 palliative care experts from 6 countries. In addition, technical piloting was carried out by 2 technical experts. The final version of the questionnaire consisted of 28 questions, constructed according to the four dimensions of the WHO ‘Public Health Model for Palliative Care’: policy, access to medicines, palliative care education and implementation of palliative care services ^19^.

The questionnaire was subdivided into eight sections:

- Consent for participation in the study (Questions 1-4)
- Demographic information about the respondent and their organisation (Questions 5-14)
- Service provision and funding for palliative care (Questions 15-18)
- Palliative care policy, legislation and ‘vitality’, captured by the existence of national strategies or plans, clinical guidelines and laws relating to palliative care, as well as the existence of a national palliative care association, regular conferences, a national journal and evidence of co-operation with other specialities (Questions 19-20)
- Access to medicines for pain relief (Questions 21-22)
- Palliative care education / training (Questions 23-25)
- Palliative care provision for children (Questions 26-27)
- Usefulness of the world map in promoting palliative care development (Question 28)

To facilitate completion of the questionnaire in a multi-lingual environment, as well as to facilitate data analysis, the majority of the questions were either multiple-choice or had pre-defined answers in a drop-down menu.

SurveyMonkey was used to host the English language questionnaire and it was anticipated that the questionnaire could be completed in approximately 20 minutes. The questionnaire was designed in a way that participants could skip questions and also pause and return to it at a later stage, in anticipation that this would reduce the number of partially complete questionnaires.

A Word version of the questionnaire was produced in March 2018 for participants with limited access to the online questionnaire. In April 2018, a professional company, ‘Language Insight’, was contracted to translate the Word version into French and Spanish in order to facilitate completion for participants in French- and Spanish-speaking countries. Completed paper questionnaires received by email were then inputted into SurveyMonkey by the research team.

The survey was opened on 20 February 2018, when an email letter of invitation containing a link to the questionnaire was circulated to contacts in the WHO regions of Africa, Eastern Mediterranean and Europe. Similar emails to contacts in WHO regions for the Americas, South-East Asia and Western Pacific were sent the following day. This two-step approach was applied to facilitate dealing with large numbers of potential queries and problems from email recipients, should these arise. In order to maximise response rate, a system of personalisation was used, i.e. participants were individually addressed in the email which was also signed by NB in order to initiate a personal relationship between researcher and participant, which we could later build on when sending out reminders. Additionally, contacts were reminded that their anonymity would be preserved in the study.

One month was given to complete the questionnaire and an Excel spreadsheet was designed to monitor incoming returns. By the end of this period, we had received questionnaires from 75 countries. For 13 countries we had received more than one questionnaire.

First reminders were circulated on 20 March 2018 and second reminders on 24 April 2018. Reminder emails, which were again personalised, stressed the importance of the study and the participant’s response to the overall project. By mid-May 2018, we had received completed questionnaires from 111 countries, with 35 countries providing more than one questionnaire. In an effort to increase response rates further, we started to keep weekly statistics and circulate weekly updates to our project partners. Contacts who had still failed to respond were contacted by DC and other team members in May, June and July 2018 and a slow increase in response rates was evident. The survey was closed on 31 August 2018, although two fully completed questionnaires were subsequently included that arrived in early September as these particular countries had been granted an extension.

***Collection of secondary data (May to December 2018).*** Secondary data were collected for countries where no contact could be identified, as well as to supplement information for countries where no complete questionnaire was returned.

To begin, data were extracted from the published atlases of palliative care for the Eastern Mediterranean and African WHO regions. Further relevant peer-reviewed items were identified through PubMed and Google Scholar using the MeSH search terms ‘palliation’ OR ‘palliative care’ OR ‘end of life care’ OR ‘terminal care’ AND ‘country’ (inserting the name for each individual country). Returns were selected using filtering by title and abstract. Information was selected from published literature according to the WHO dimensions and the 10 indicators described below.

Inclusion criteria were: reference to at least one of the dimensions of the WHO palliative care public health strategy (education, policy, implementation of palliative care services, medicine availability) plus vitality / country-level data / published in English, French, German or Spanish between 2014 and 2018 and not reliant on data published in any of the regional palliative care atlases or the previous two world maps.

In addition to peer-reviewed items, ‘grey literature’ identified on the internet was used to fill gaps, including governmental websites, ehospice, EAPC blogs, conference presentations and policy statements.

### Data entry, cleansing and storage

Using SurveyMonkey as an online survey tool meant that most data arrived in electronic form, but 23 questionnaires were returned by email. Of these, 15 were English language questionnaires, four were French and four were Spanish. In order to facilitate data analysis, these were printed and entered into SurveyMonkey by the research team at the University of Glasgow. Afterwards, the printed copies of these questionnaires were stored in a locked cabinet in the PI’s office.

Once the survey closed, all data were downloaded into an Excel spreadsheet and cleansed for accuracy and completeness. As the number of responses was manageable, this task was carried out manually at the University of Glasgow. Cleansing included the removal of duplicate responses which resulted from participants abandoning a questionnaire and starting a new one after receiving a reminder email as well as checking for ‘illegal values’, i.e. values that were outside the definition of accepted values. In a further step, any missing values were flagged for completion with supplementary data from other sources at a later stage. Special attention was given during the cleansing process to data that had been entered from paper questionnaires, as respondents frequently presented information that could not be captured in the online system and which did not meet the entry criteria but had to be entered manually into the Excel spreadsheet.

After the cleansing process, every record was given a code before personal data were removed from the spreadsheet and stored separately in a password protected file. The file containing the research data was also stored on a password protected computer and accessible only to the primary researcher at the University of Glasgow.

### Data analysis

Data analysis started in September 2018 and priority was given in the first instance to the survey returns.

**Figure 2.**
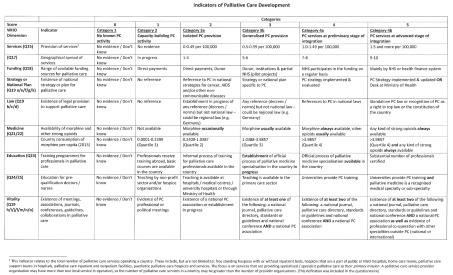
Indicators of Palliative Care Development.

***a) Analysis of data obtained from questionnaires***. Data analysis built on a scoring system developed by the research team. Based on the four dimensions detailed in the WHO ‘Public Health Model for Palliative Care’, the following table of ten qualitative and quantitative indicators had been created collaboratively in November 2017 (see Figure 2), with each of the indicators relating to one or more questions/sub-questions. The final questions had thus been shaped by the agreed indicators, shown in Table 2.

**Table 2.** Relation of indicators in the questionnaire to the WHO ‘Public Health Model for Palliative Care’.

WHO Dimension	Indicator of choice	Question(s)
Implementation	Provision of services per 100,000 population	15
	Geographical spread of services	16
	Source(s) of funding	18
Policy	Strategy / national plan	19a/e/f/g/k
	PC law	19b/c/d
	Vitality	19h/i/j/l/m/n/o
Access to medicine	Availability of opioids	21/22
	Opioid consumption (mg per capita)	data from International Narcotics Control Board ^iii^
Education	Training for professionals	23
	Education for future doctors/nurses	24/25

^iii^Boardprovided by Professor James Cleary

Each indicator was then assigned a range of characteristics representing the six categories used in the second world map, now expanded, as shown in Table 3.

**Table 3.** Expanded categories of palliative care development (2018).

**Category 1:** No known palliative care activity	A country in this category is one where current research reveals no evidence of any palliative care activity.
**Category 2:** Capacity-building palliative care activity	A country in this category shows evidence of wide-ranging initiatives designed to create the organisational, workforce and policy capacity for the development of palliative care services, although no service has yet been established. Developmental activities include attendance at, or organisation of, key conferences, personnel undertaking external training in palliative care, lobbying of policy makers and Ministries of Health and emerging plans for service development.
**Category 3a:** Isolated palliative care provision	A country in this category is characterized by the development of palliative care activism that is still patchy in scope and not well-supported; sources of funding that are often heavily donor-dependent; limited availability of morphine; and a small number of palliative care services that are limited in relation to the size of the population.
**Category 3b:** Generalised palliative care provision	A country in this category is characterized by the development of palliative care activism in several locations with the growth of local support in those areas; multiple sources of funding; the availability of morphine; several hospice-palliative care services from a range of providers; and the provision of some training and education initiatives by the hospice and palliative care organizations.
**Category 4a:** Palliative care services at a preliminary stage of integration to mainstream health care services	A country in this category is characterized by the development of a critical mass of palliative care activism in a number of locations; a variety of palliative care providers and types of services; awareness of palliative care on the part of health professionals and local communities; a palliative care strategy that has been implemented and is regularly evaluated; the availability of morphine and some other strong pain-relieving drugs; some impact of palliative care on policy; the provision of a substantial number of training and education initiatives by a range of organizations; and the existence of a national palliative care association.
**Category 4b:** Palliative care services at an advanced stage of integration to mainstream health care services	A country in this category is characterized by the development of a critical mass of palliative care activism in a wide range of locations; comprehensive provision of all types of palliative care by multiple service providers; broad awareness of palliative care on the part of health professionals, local communities, and society in general; a palliative care strategy that has been implemented and is regularly updated; unrestricted availability of morphine and most strong pain-relieving drugs; substantial impact of palliative care on policy; the existence of palliative care guidelines; the existence of recognized education centres and academic links with universities with evidence of integration of palliative care into relevant curricula; and the existence of a national palliative care association that has achieved significant impact.

In order to facilitate quantitative analyses and categorisation of countries, a scoring matrix was created, assigning each indicator a value between 0 (Category 1) and 5 (Category 4b). Pilot scoring and moderation of three countries took place between the Glasgow team and ATLANTES and subsequently, the scoring matrix was circulated again to the collaborators for feedback in May and June 2018.

When starting the analysis, priority was given to countries where only one completed questionnaire was available. For countries with more than one completed questionnaire that showed discrepancies in the responses, the above-mentioned algorithm of sampling key experts was applied to decide on an ‘index questionnaire’ for this country that would form the basis of the analysis. Information obtained from additional questionnaires from this country was then used as complementary material where required. Table 4 shows the number of questionnaires returned by country.

**Table 4.** Number of returned questionnaires per country.

No of returned questionnaires per country	No of countries
Countries with 1 questionnaire	98
Countries with 2 questionnaires	59
Countries with 3 questionnaires	20
Countries with 4 questionnaires	1
Countries with 5 questionnaires	0
Countries with 6 questionnaires	1
Countries with 7 questionnaires	1

**Figure 3.**
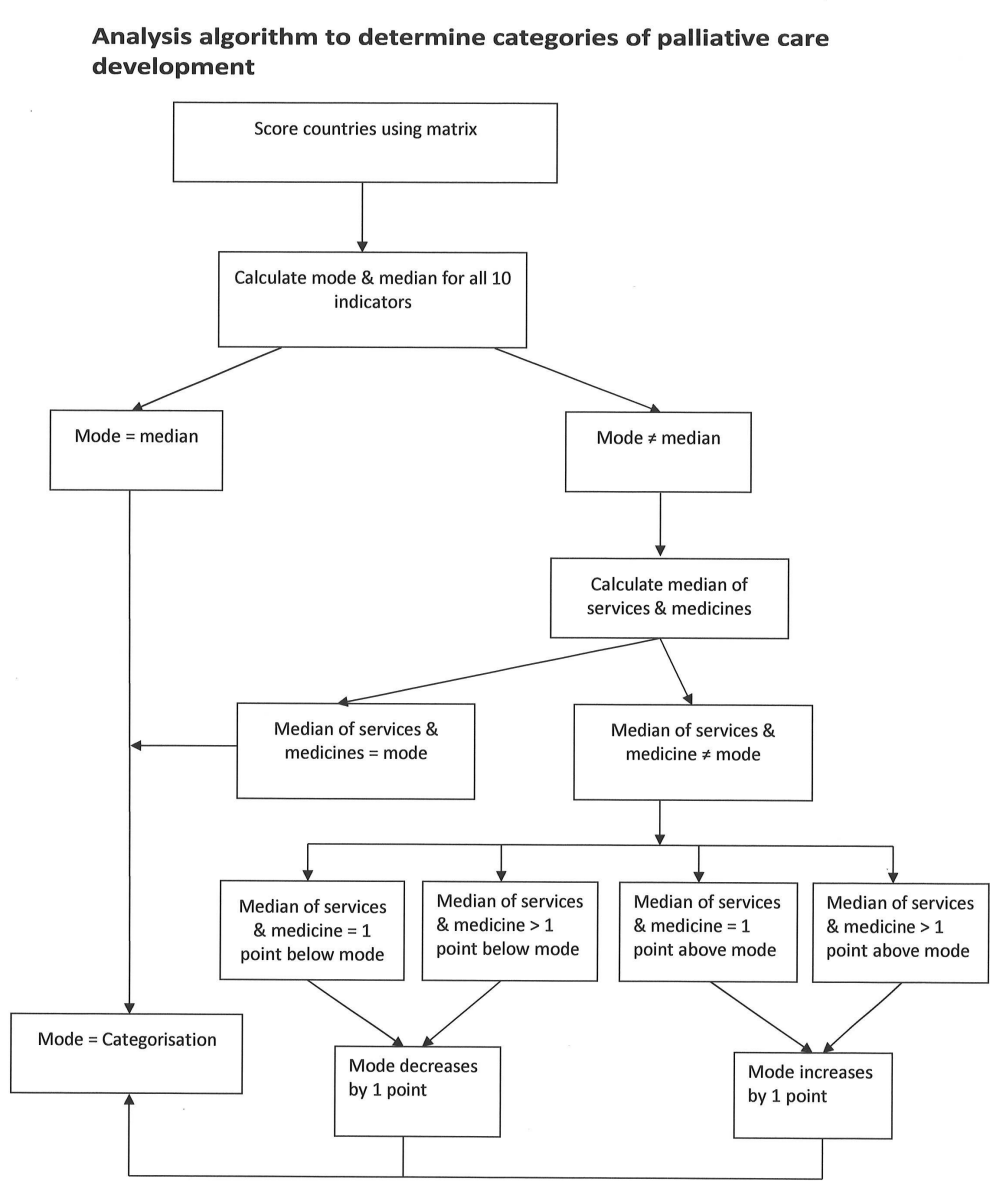
Data sourcing process.

**Figure 4.**
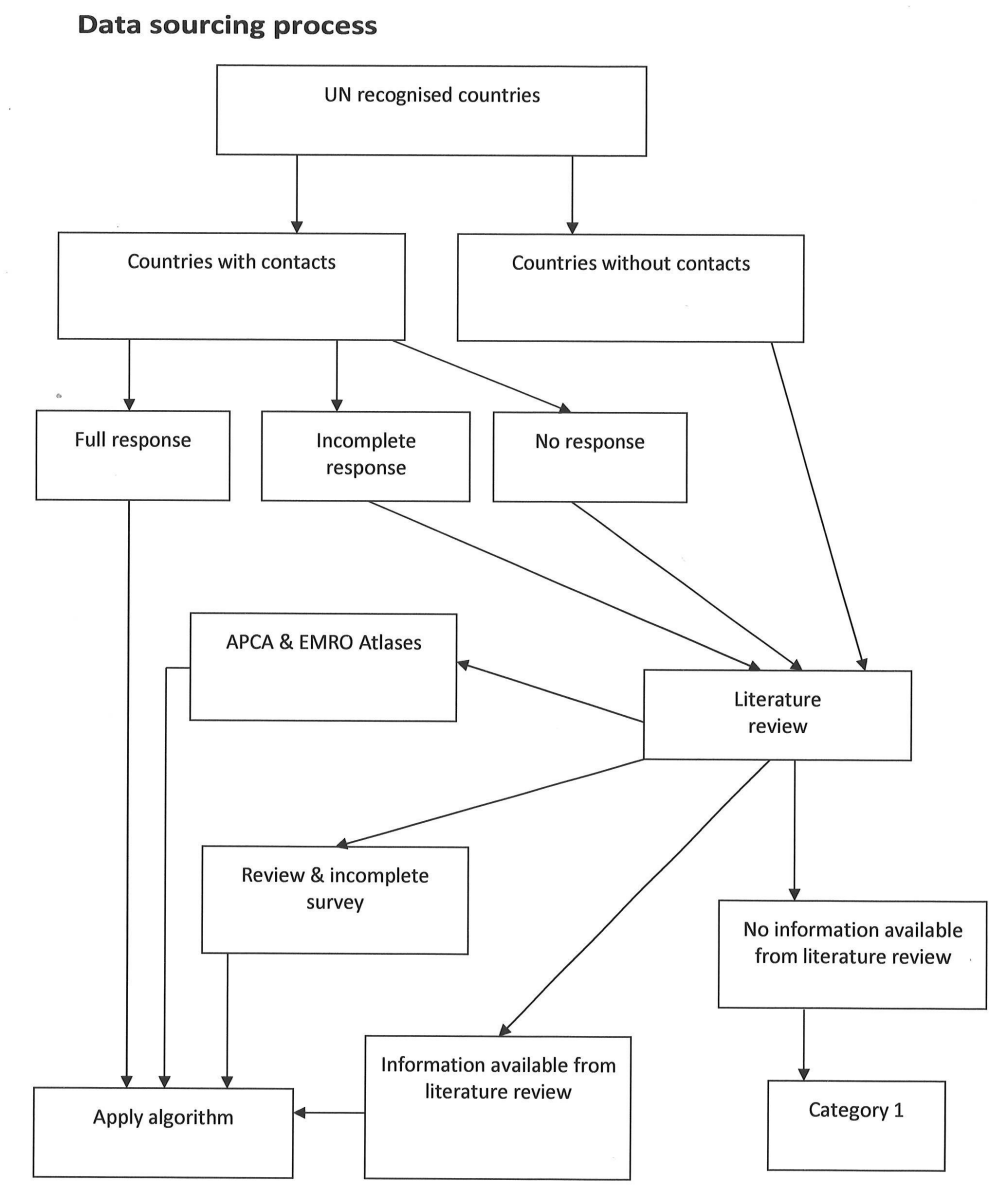
Algorithm determining WM3 categories.

Countries from which we received at least one fully completed questionnaire were processed through the algorithm in Figure 4. As shown in Figure 3 the countries where no contact respondent had been identified, which provided incomplete questionnaires or failed to respond to our reminders went into a review process involving the research team. For these countries, missing data were supplemented by information gathered from published literature, including the most recent regional atlases for the African (APCA) and Eastern Mediterranean (EMRO) regions. If sufficient information could be gathered in this way, these countries proceeded to the above-mentioned algorithm. If only insufficient or no information at all could be identified, the countries were assigned to category 1.

An algorithm was developed to establish the level of palliative care development for each country in the study ( Figure 4). The algorithm was applied to all countries for which sufficient data could be gathered from either the questionnaires alone or the questionnaires and supplementary secondary sources combined. The mode of the 10 indicators, the most repeated value, and the median, the measure of central tendency, were then used to determinate the level of development for each country as follows:

a) When the mode and median of the 10 indicators were coincident, the most repeated value was also in the middle of the distribution of the 10 values. This then determined the country’s level of categorization of palliative care. 

b) When the mode and the median were not coincident, we took the categorical view that the most significant indicators for judging palliative care development are the existence of essential medicines for pain control and the provision of palliative care services. In our survey, we employed two indicators on medicines (concerned with the availability and consumption of opioids) and two indicators on services provision (the total number of services per population and the distribution of these services). We therefore included these four indicators in the algorithm as the ‘most consequential indicators’ of palliative care development. Thus:

i. When the mode of the 10 indicators was coincident with the median of the most consequential indicators, that number was used to determine the level of categorization, meaning that the most consequential indicators (services and medicines) were jointly coincident with the most repeated position of the other indicators.

ii. When the mode was to the left-hand side of the median of the most consequential indicators (i.e. the mode was less than the median for services and medicines), then the indicators for services and medicine were used to moderate the level by taking the mode slightly to the right and assigning mode + 1 as the level of development.

iii. When the mode was to the right-hand side of the median of the most consequential indicators (i.e. the mode was greater than the median for services and medicines), then the indicators for services and medicine were used to moderate the level by taking the mode slightly to the left and assigning mode -1 as the level of development.

***b) Third party sources***. The analysis relied, for some items, on third-party information. In order to calculate the number of services per 100,000, for example, population figures from the 2017 World Population Data Sheet were used. Data regarding the per capita opioid consumption were obtained by DC from the University of Wisconsin Pain and Policies Study Group through personal communications with Professor James Cleary (now of the Walther Supportive Oncology Program at Indiana University School of Medicine).

***c) Analysis of secondary data***. Analysis of secondary data was applied to countries with no contact respondent or countries where contacts failed to reply and was used to supplement information for countries which returned only a partially complete questionnaire. Although we searched for secondary data to match the indicators as closely as possible, the nature of such information made it impossible to include four of these countries in the scoring matrix. Instead, they were assigned a world map category by the research team, based on expert judgement and contextual knowledge.

Most of the data analysis was completed from September to November 2018 by the research team in Glasgow, where regular moderation meetings between the researcher and the PI ensured accuracy and consistency. Further moderation took place with ATLANTES, which culminated in a face-to-face meeting in Dumfries in December 2018. In a small number of cases, respondents had to be contacted again in order to clarify answers.

### Ethical considerations

The study was subject to review by the University of Glasgow College of Social Sciences Research Ethics Committee. Approval was granted on 15 January 2018 (Application No: 400170065). Prior to data collection, the purpose of the study was explained to all participants and written consent for participation in the study was obtained from each participant before access to the questionnaire was granted. Participants were informed that they had the right to withdraw from the study at any point in time, without any repercussions.

### Confidentiality and data protection

Paper questionnaires were stored in a secure location at the University of Glasgow. All electronic research data were stored as de-identified data (i.e. identifiers have been replaced by a code) in a password protected file.

Personal data on the respondents were collected for administrative purposes. As soon as the questionnaires were returned, the respondents were assigned a unique code that was linked to the respondent in a password-protected Excel database and will not be used in any publications. The Excel database was stored separately from the questionnaire and personal data will not be disclosed either orally or in writing to any unauthorised party. Personal data will only be retained for the length of the data collection period. Afterwards the electronic file containing personal data will be permanently deleted.

Participants were made aware that a copy of the final manuscript of their data is available to them upon request.

### Data dissemination and resource sharing

Following the wide interest and high citation levels of publications about the first two world maps of palliative care development, it is anticipated that interest in the results of this study will be high. Our publication and dissemination strategy include:

1. The current paper detailing the study protocol and methods used.
2. A ‘headline’ paper on the third iteration of the ‘world map of palliative care development’, supported by the current paper as an account of the protocol and methods.
3. A paper specifically focussed on the global development of children’s palliative care.
4. A paper on the linear ranking of palliative care development.
5. Contribution to material for inclusion in the 2 ^nd^ edition of the Global Atlas of Palliative Care at the End-of-Life. The Global Atlas includes summary results of the mapping as part of a larger publication depicting all aspects of palliative care globally. Being a co-publication with the WHO, it receives a high level of attention; the first edition has been downloaded over 70,000 times.
6. Findings from the study and related commentary will also be available on social media, including the Glasgow End of Life Studies Group blog, ehospice.com, and the project website - https://www.gla.ac.uk/research/az/endoflifestudies/projects/worldmapofpalliativecare/

ArcGIS, Version 10.5, was used to create the map for this study. In addition, a special geoprocessing tool was added to ArcMap in order to create cartograms according to the Gastner-Newman method. This technique allowed us to generate maps which make countries visually comparable by modifying their geographical size according to our specific data.

### Strengths and limitations

Figure 1 lists our summary of commentaries and concerns about the methodology of the previous world map studies, together with our responses to these issues in the present study.

The present study suffers from potential biases associated with key informants. Such self-reported data cannot be independently verified and we are aware that some respondents might have deliberately underrated or overrated levels of development in their country for their own reasons. For countries where more than one questionnaire was returned, the research team had to moderate discrepancies between the answers. Although the use of key informants from the field of palliative care has its drawbacks, we are not convinced that ‘government sources’ can necessarily provide a more detailed report of palliative care provision in a given country and we remain committed to the use of in-country specialists, who by their involvement also help to build capacity for the wider effort of palliative care mapping.

In some instances, we were unable to identify two palliative care experts in-country and in other countries, where only one eligible person was identified, they failed to complete the questionnaire. Data limitations were compounded by language constraints. Questionnaires were only available in three European languages. Whilst this impeded communication with reluctant respondents, it might also have added to the barriers for non-native speakers willing to complete the questionnaire. Furthermore, not all data provided in the questionnaires relates to the year 2017 and, as the research team was reliant on third-party data for some information, some minor data gaps exist. 

### Study status

At the time of publication of this article, all data for the study has been collected and analysed and all visuals have been prepared. The study is currently unpublished with a draft article in preparation.

## Conclusions

The first and second ‘world maps’ of palliative care development created in 2006 and 2011 have become important tools for presenting the global process of palliative care development in the world through an international comparative analysis. Allocating each country to one of six levels of palliative care development has enabled us to chart progress over time.

The third world map will allow us to assess global palliative care development over the period 2006-17. Taken together, the three maps will form a valuable tool for advocacy, in a context where the study results are relevant to governments, policy makers, palliative care activists, service providers and the wider public.

The ultimate beneficiaries of increased understanding and measurement of palliative care development are the patients and families that need palliative care but are unable to access the care they require. The enormous unmet need for palliative care, particularly in low and middle-income countries, is a largely unrecognized matter of public health urgency. This work will help to highlight the continuing gaps that exist in palliative care development around the world.

## Data availability

### Underlying data

No underlying data are associated with this article.

### Extended data

Enlighten: Research Data: Recalibrating the 'world map' of palliative care development. https://doi.org/10.5525/gla.researchdata.779 ^18^

This project contains the following extended data:

- Questionnaire.pdf (Copy of online consent and questionnaire form)
- World_map_of_palliative_care_development_drop_down_menus (Drop-down answer options for sections 3, 4 and 5 of the questionnaire)
- Participant_Information-World Map_revised.pdf (Participant information sheet)

Data are available under the terms of the Creative Commons Attribution 4.0 International license (CC-BY 4.0).
