# Supplementary material for: Recalibrating the ‘world map’ of palliative care development
Source: Wellcome Open Res. 2019 Aug 16;4:77. Originally published 2019 May 2. [Version 2] doi: 10.12688/wellcomeopenres.15109.2 (PMC6556994; doi:10.12688/wellcomeopenres.15109.2)
Supplement: Peer review history

# Peer review: Reviewer response for version 1

**DOI:** 10.21956/wellcomeopenres.16485.r35434

**Published:** 2019-06-04

**Authors:** Philip Larkin

**Affiliations:** 1 Lausanne University Hospital (CHUV), Faculty of Biology and Medicine, University Institute of Training and Research in Patient Care (IUFRS), University of Lausanne, Lausanne, Switzerland

This is an important paper written by a group with strong credibility in the field of palliative care and with regard to this level of global mapping. It is useful to have a 'methods' paper as this is often overlooked in the haste to present the data and in fact, this paper demonstrates the complexity of such an undertaking. It is largely well written but I would suggest an overall review for gammar and spelling. I note, for example, on pg 12 that the tense moves from the past to the future and I believe that if indexed, it should be written in the past tense as far as possible. 

 I do wonder if pg 12 (Information leaflet) adds to this paper and unless essential to the discourse, I would suggest it is either excluded or placed as an appendix. I am not sure what added value it brings. 

 The scoring system is complex and rather well explained. I think the algorithms would benefit from some style editing. They are clear but perhaps could be presented in a better visual format. One comment is noted on page 6 where the development of the questionnaire refers to the possibility to 'skip questions' but it is not clear to me if this was designed initially (which I thought on pg 5) but seems to have been an adaptation later. I think that could be clarified. 

 Other than these minor points, I would commend the authors for a comprehensive and well proportioned paper.

I confirm that I have read this submission and believe that I have an appropriate level of expertise to confirm that it is of an acceptable scientific standard.

# Peer review: Reviewer response for version 1

**DOI:** 10.21956/wellcomeopenres.16485.r35435

**Published:** 2019-05-24

**Authors:** Liz Grant

**Affiliations:** 1 Global Health Academy and the Primary Palliative Care Research Group, the Usher Institute, University of Edinburgh, Edinburgh, UK

This is the third world map of palliative care. It builds on two previous ones (2006 and 2011) developed by Clark and colleagues introducing a more complex and comprehensive analysis of the reach, quality and coverage in each country.

 The first map provided a basic matrix looking at four categories: No identified hospice-palliative care activity; Capacity building activity but no service; Localized palliative care provision; Palliative care activities approach integration with mainstream service providers. The second map,  recognising the gaps in the data collection of the 1 ^st^ process especially with regards to reliance on published literature, worked with in-country experts who commented on the level of palliative care engagement using a refined set of six categories which included the following: No known hospice-palliative care activity; Capacity-building activity; Isolated palliative care provision; Generalized palliative care provision; Countries where hospice-palliative care services are at a stage of preliminary integration into mainstream service provision; Countries where hospice-palliative care services are at a stage of advanced integration into mainstream service provision.

 This third mapping project has sought to improve the rigour of the methods of data collection, and the quality of data collected

 It had the following objectives:

1. To establish the level of palliative care development in UN countries in 2017.
2. To implement methodological improvements, based on recent progress in the field and commentaries relating to the previous ‘world map’ studies.
3. To monitor development in global palliative care since 2006 and 2011.

The work was extensive in its analysis. Recognising the value of experts a new database was established of experts who were able to provide a much more nuanced summary of the state of palliative care in their country. The study also linked to the various networks and Associations of Palliative care to increase the number of people with considered opinions on palliative care.

 An algorithm was established to enable sampling to take place with a list of preferred categories

 These included four separate categories:   

1. Representatives of the national in-country hospice-palliative care association or nearest professional association
2. Academic experts with research experience in hospice-palliative care development in-country
3. Government and Civil Society Policy specialists with knowledge of palliative care
4. Palliative care representatives, academics or policy specialists from outside the country who have knowledge of the country

Data was collected using an online questionnaire (In English speaking countries this was Survey Monkey – no data given of what it was in other countries) in 179 UN countries.

 No experts could be located in 19 countries. 560 people were interviewed. Information about  these categories is very important as these sources are the ones who have shaped the constructs. 

 Data on the total country population and per capita opioid consumption were derived from a set of different independent sources. More information on these would be helpful.

 Interview data was analysed using a scoring system developed by the researchers. This leads to a minor limitation which the authors identified.  A weighting system was also developed by the group.

 Other limitations that the authors identified were the challenges of self reported summaries especially when experts were involved in the setting up and development of country services and where their future income form external donors depended on good results, internal moderation of the discrepancies  between different country perspectives.

 The authors do note that though there are most certainly challenges in relying on country experts because of their biases they noted that ‘government sources’ could also be unreliable – sometimes out of data, capturing urban public sources.  Data is only as good as the carefulness of those who have inputted it and the quality of what was inputted.   

 Further limitations were that the questionnaires were only available in three European languages and this could have significantly affected the feedback and the accuracy of the feedback from those who only spoke  local languages.

 There is also concern about the years that the data relate to – the respondents note that not all countries had data that went up to 2017 so there is a danger that comparisons across are false.

 More information is needed on the comprehensiveness of the academic expert conversations especially as a number of academics in countries expressed a lack of awareness about the 1 ^st ^and 2 ^nd^ maps and about the processes.

 However despite the multiple challenges the map has provided a very important step forward in

 a) demonstrating that we need to understand coverage, b) in profiling the importance of multi country analysis of palliative care in a way that corresponds to the effort and significance that other diseases and other health system services such as maternal health, have had.

 The three maps demonstrate the changes in societal understanding of palliative care in different countries. This work will help to highlight the continuing gaps that exist in palliative care development around the world. As such it is an exceptionally important piece of work.

I confirm that I have read this submission and believe that I have an appropriate level of expertise to confirm that it is of an acceptable scientific standard.
